# Integrated Histological, Ultrastructural, and Transcriptomic Analyses Reveal New Insights into Stamen Development in Cytoplasmic Male Sterile Tobacco (CMS K326)

**DOI:** 10.3390/plants14172613

**Published:** 2025-08-22

**Authors:** Fangfang Cui, Shanshan Qiu, Jikai Ma, Yanbo Wang, Jiange Wang, Qiyuan Liu

**Affiliations:** 1 College of Agronomy, Jiangxi Agricultural University, Nanchang 330045, China; cff@stu.jxau.edu.cn (F.C.); qiyuanl@126.com (Q.L.); 2 School of Forestry, Jiangxi Environmental Engineering Vocational Institute, Ganzhou 341000, China; s3a3n@126.com; 3 College of Forestry, Jiangxi Agricultural University, Nanchang 330045, China; jkma@jxau.edu.cn; 4 School of Soil and Water Conservation, Nanchang Institute of Technology, Nanchang 330099, China; 2019994704@nit.edu.cn

**Keywords:** *Nicotiana tabacum*, cytoplasmic male sterility (CMS), mitochondria, auxin, stamen

## Abstract

Cytoplasmic male sterility (CMS) is a crucial tool for exploiting plant heterosis, though its underlying mechanisms in tobacco remain incompletely understood. In this study, Tobacco CMSK326, derived from a naturally occurring variant of *Nicotiana tabacum*, exhibited a range of stamen abnormalities, including stamen degeneration, stamen absence, and carpelloid, petaloid, and sepaloid traits. Histological and electron microscopy showed that CMS K326 and its maintainer differentiated at the early bud stage. Analysis of differentially expressed genes (DEGs) revealed abnormal expressions of several key genes, including *WUSCHEL* (*WUS*), *GLOBOSA* (*GLO*), *SUPERMAN* (*SUP*), and auxin-related genes such as *AUX22*, during bud development. Weighted gene correlation network analysis (WGCNA) identified a module highly correlated with flower development, the tricarboxylic acid cycle (TCA), auxin, and the mitochondrial retrograde regulation signal molecules *CDKE1* and *KIN10*. The promoter regions of 19 out of the 42 hub genes in this module possess auxin cis-response elements. These results point to a correlation between auxin irregularities and stamen development in CMS K326.

## 1. Introduction

Cytoplasmic male sterility (CMS) is a phenomenon in which flowering plants cannot produce functional pollen while the pistil remains normal, showing maternal inheritance. CMS displays variations in stamens, such as aborted pollen and deformed (degenerate anthers, degenerate filaments, and absent stamens), carpelloid, petaloid, and sepaloid stamens [1]. The phenomenon of carpelloid, petaloid, and sepaloid stamens in CMS resembles the flower homeosis regulated by nuclear genes, commonly known as cytoplasmic homeosis [2].

CMS is a crucial tool for utilizing plant heterosis, yet its mechanismremains unclear [3]. When explaining the mechanism of CMS, it is necessary to address where and when it starts. CMS with pollen abortion typically occurs in the late stages of microspore formation, whereas CMS with carpelloid, petaloid, and sepaloid stamens appear during the early stages of flower development [4]. The latter is often accompanied by the disappearance of the boundary between whorls 3 (stamens) and whorls 4 (pistils) [4]. Studies conducted on several crops showed that the arrangement of the mitochondrial genome could lead to CMS by generating new open reading frames (ORFs) or chimeric ORFs containing coding genes associated with the mitochondrial function [4,5,6]. However, it should be noted that not all studies on CMS support this conclusion, as some assumed ORFs linked to CMS cannot be attributed to the CMS phenotype [1,7]. The study found a rearrangement of mitochondrial DNA in tobacco CMS, but no new or chimeric ORF was observed [8]. Not only were mitochondrial DNA rearrangements observed in CMS with carpelloid, petaloid, and leaf-like stamens, but there was also a down-regulation in the expression of B or C genes [2]. At the same time, the expression of the cadastral gene *SUPERMAN* (*SUP*) was also down-regulated. However, their order of flower whorls remained unchanged compared with the maintainer [9]. This suggested that floral organ identity genes can change their organ characteristics but have little effect on their development [1]. Therefore, CMS stamens with cytoplasmic homeosis might be caused by interference in the expression of floral organ identity genes by the corresponding floral organs during differentiation. *SUP* studies have revealed that abnormal stamens result from excess stem cell proliferation in the fourth whorls rather than the mutual transformation between male and female stamens [10]. According to the abovementioned results, it appears that stamen primordial stem cells did not initiate correctly while the CMS flowers’ organogenesis took place. Similarly, pistil primordial stem cells did not terminate correctly during the flower organs’ differentiation [10]. Thus, as a result of cellular activity disorder, stamens of CMS become abnormal [10].

Tobacco (*Nicotiana tabacum*) is an important economic crop worldwide. Based on prior evidence that mitochondrial dysfunction alters auxin transport in CMS systems [11,12], we hypothesized that CMS K326 stamen defects arise from stage-specific auxin signaling disruption caused by mitochondrial retrograde regulation. To test this, we designed an integrated histological, ultrastructural, and transcriptomic study with three objectives: (i) Define the developmental window of stamen abnormality initiation using histology/transmission electron microscopy (TEM). (ii) Characterize mitochondrial ultrastructural anomalies correlated with meristem defects. (iii) Decipher the regulatory network linking mitochondrial dysfunction to auxin-responsive floral gene networks. Our integrated analysis revealed a link between abnormal auxin levels and stamen development within CMS K326 plants.

## 2. Results

### 2.1. The Stamens of CMS K326 Differed from Those of the Maintainer in the Small-Bud Stage (<3 mm)

**Figure 1:**
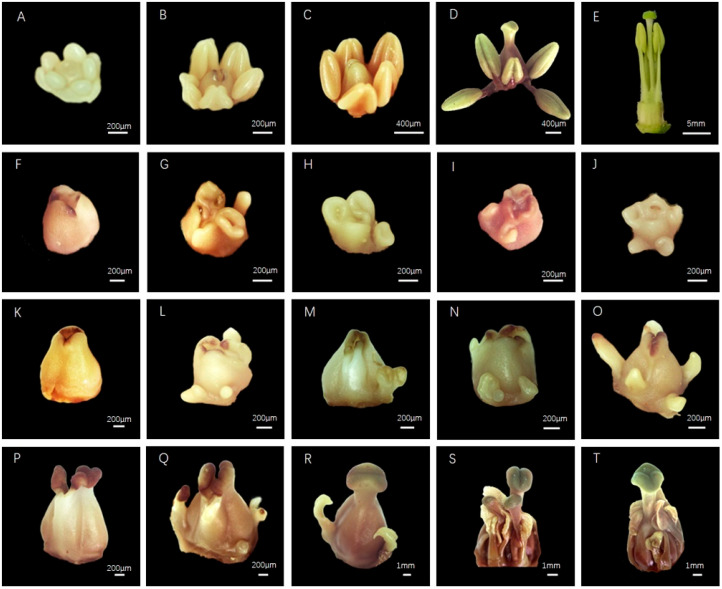
The stamen morphologies of CMS K326 and its maintainer in different bud stages. (**A**–**E**) The maintainer buds. (**F**–**J**) CMS K326 buds in the small-bud stage (<3 mm). (**K**–**O**) CMS K326 buds in the medium-bud stage (3–5 mm). (**P**–**T**) CMS K326 buds in the big-bud stage (>5 mm).

Observations of the flower buds of CMS K326 at various stages and its maintainer revealed developmental differences in stamens (Figure 1). During the small-bud stage (<3 mm), the stamens were still developing (Figure 1A,F–J). The stamens of CMS K326 were aberrant, showing traits such as stamen loss, polymorphic separation, fusions at the apex, fusions at the base, and a few separated stamens (Figure 1F–J). Fusions at the stamen base showed the boundary damage between the third and the fourth flower whorls. In this stage, the maintainer’s stamens stayed entirely apart, the anthers formed, and the tips of the stamens continued to develop (Figure 1A). Stamens differentiated significantly in the medium-bud stage (3–5 mm). Certain CMS K326 stamens resembled stigmas, while others transformed into leaflike and petaloid structures (Figure 1K–O). In contrast, the maintainer showed fully developed anthers at the top (Figure 1B). The maintainer’s styles were separated into two lobes, and simultaneously, some CMS K326 stamens elongated along with the pistils, forming pistils with 3–5 lobes (Figure 1R–T). Within CMS K326, certain stamens regressed at a specific stage and were shorter than the height of the pistil. (Figure 1S–T). Ultimately, CMS K326 showed the characteristics of stamen petaloid, carpelloid, sepaloid, and degeneration, which led to the inability to form normal stamens and produced functional pollen. The morphological observations showed that the abnormal stamens appeared during the initial bud development phase, suggesting that stamen dysfunction might have started before this stage.

**Figure 2:**
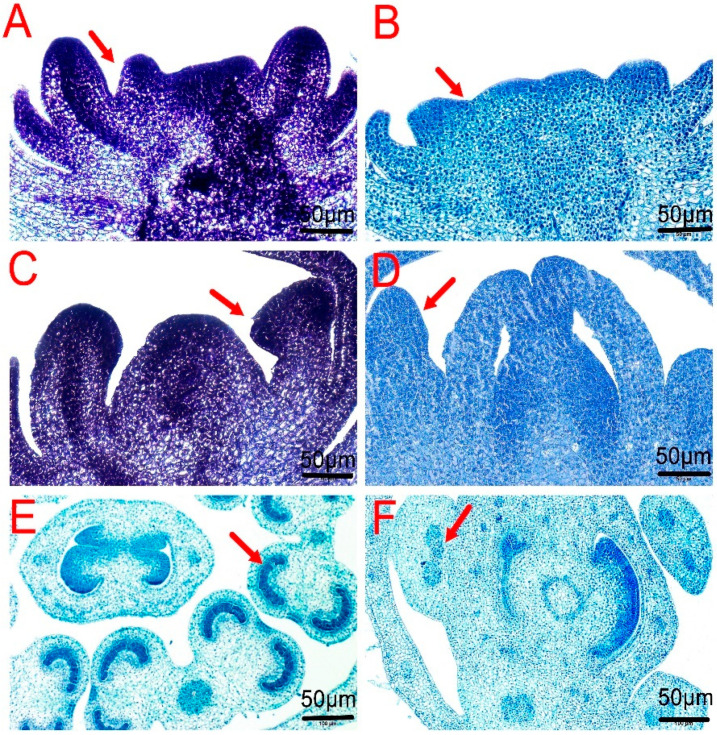
The stamen development in the CMS K326 and its maintainer in different bud stages. (**A**) The stamen of the maintainer in the small-bud stage. The arrow shows a clear boundary between the pistils and the stamens. (**B**) The stamen of the CMS K326 in the small-bud stage. The arrow shows that the boundary between the pistils and the stamens has disappeared. (**C**) The stamen of the maintainer in the early middle-bud stage. The arrow shows that the stamens have differentiated. (**D**) The stamen of CMS K326 in the early middle-bud stage. The arrow shows an undifferentiated stamen. (**E**) The stamen of the maintainer in the late middle-bud stage. The arrow shows that the pollen sacs have differentiated. (**F**) The stamen of CMS K326 in the late middle-bud stage. The arrow shows undifferentiated anther sacs.

Morphological observations suggested that the dysfunction of stamens in CMS K326 precedes the formation of small buds. To further elaborate on CMS K326’s stamen abnormality position and stage, the flower buds of CMS K326 and its maintainer were compared at different bud stages using histological sections (Figure 2). During the small-bud stage in the maintainer, the calyxes underwent development, and the petals and stamens began forming on the meristem’s side. This led to the formation of the second and third whorls, respectively. Moreover, the center of the meristem appeared flat while the carpel was developing (Figure 2A). Meanwhile, the CMS K326 carpel primordium and stamen primordium were fused, making the boundary between them less noticeable (Figure 2B). At the middle-bud stage, the maintainer stamens were level with or above the pistil, with clear differentiation at the top (Figure 2C). In contrast, CMSK326 stamens were undifferentiated (Figure 2D). In the late middle-bud stage, the maintainer’s anther morphology was established, characterized by four separate pollen sacs resembling a butterfly (Figure 2E). Nonetheless, the anthers in CMS K326 were malformed or undifferentiated (Figure 2F). The tissue sections mentioned above showed that stamen dysfunction occurred in the meristem stage of CMS K326.

**Figure 3:**
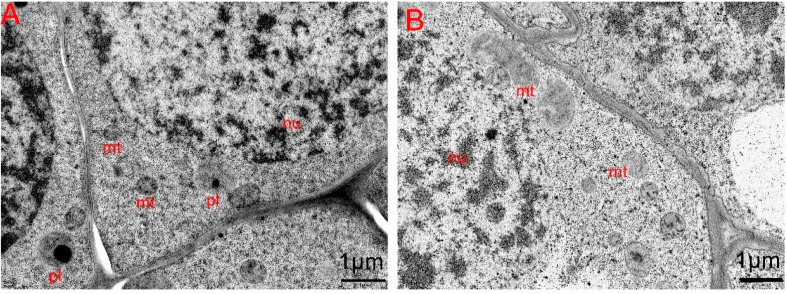
Electron microscopic observation of floral meristem in CMS K326 and its maintainer. (**A**) The maintainer. (**B**) CMS K326. nu: nucleus. mt: mitochondria. pl: plastid.

Using an electron microscope, it was observed that the mitochondria in the maintainer’s flower meristem had a uniform morphology (Figure 3A). In contrast, the flower meristem of CMS K326 exhibited a disorganized arrangement of mitochondrial cristae. These abnormal mitochondria exhibited apparently enlarged morphology compared to adjacent normal mitochondria in CMS cells (Figure 3B, upper vs. lower), with disrupted cristae architecture.

In conclusion, CMSK326 showed differences from its maintainer during the meristem stage.

### 2.2. Analysis of Differentially Expressed Genes (DEGs) During the Three Stages of Bud Development

Eighteen samples, including CMS K326 and its maintainer at three different bud stages, underwent transcriptome sequencing to reveal the molecular mechanism behind the abnormal stamen. The sequencing generated 1846,668,458 high-quality clean reads (Q30 > 94.19%), with each sample contributing at least 10.89 G. The clean reads were uniquely aligned with the reference tobacco genome TN90 (GCF_000715135.1) [13], with an efficiency ranging from 95.22% to 96.33% (Table 1). The reference-guided assembly of the mapped reads using the Cufflinks/Cuffmerge pipeline identified 72,780 genes, of which 9113 (12.52%) were novel. At least 7881 genes in each sample displayed differential expression (FPKM > 1) (Table 1).

**Figure 4:**
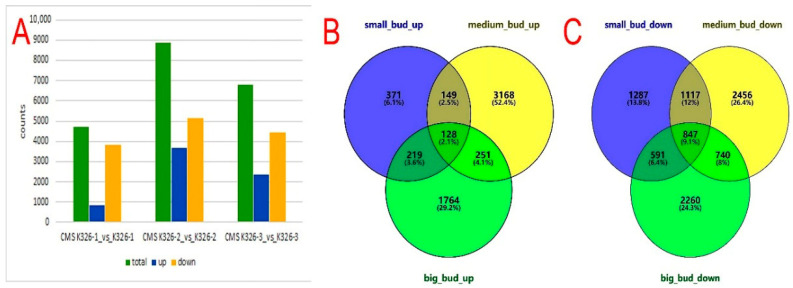
The DEGs were identified from a total of 18 samples, and Venn diagrams of DEGs among the three different bud stages. (**A**) The DEGs were identified from 18 samples. CMS K326-1_vs_K326-1: DEGs in the small-bud stage. CMS K326-2_vs_K326-2: DEGs in the medium-bud stage. CMS K326-3_vs_K326-3: DEGs in the big-bud stage. (**B**,**C**) The Venn diagram of DEGs among the three bud stages. up: up-regulated genes. down: down-regulated genes.

During the various developmental stages of small, medium, and big buds, 4709, 8856, and 6800 DEGs were identified, respectively. At the small-bud stage, a total of 867 DEGs were found to be up-regulated. Among these, 567 were known genes, while 300 were novel. Additionally, 3842 DEGs were identified as down-regulated, with 3405 known genes and 437 novel genes. Moving on to the medium-bud stage, 3696 DEGs were up-regulated, consisting of 3176 known genes and 520 novel genes. Furthermore, 5160 DEGs were down-regulated, including 4385 known genes and 775 novel genes. Lastly, in the big-bud stage, 2362 DEGs were up-regulated, with 1703 known genes and 659 novel genes. On the other hand, 4438 DEGs were down-regulated during this stage, comprising 3995 known genes and 443 novel genes (Figure 4A). qRT-PCR results are shown in Supplementary Materials Figure S1.

In total, 277 up-regulated genes were common (4.6%) between the small- and medium-bud stages, 379 up-regulated genes were common (6.2%) between the medium- and big-bud stages, and 128 up-regulated genes were common (2.1%) across all three stages. Moreover, 1964 down-regulated genes were common (21.1%) between the small- and medium-bud stages, 1587 down-regulated genes were common (17.1%) between the medium- and big-bud stages, and 847 down-regulated genes were common (9.1%) among all three stages (Figure 4B,C).

**Figure 5:**
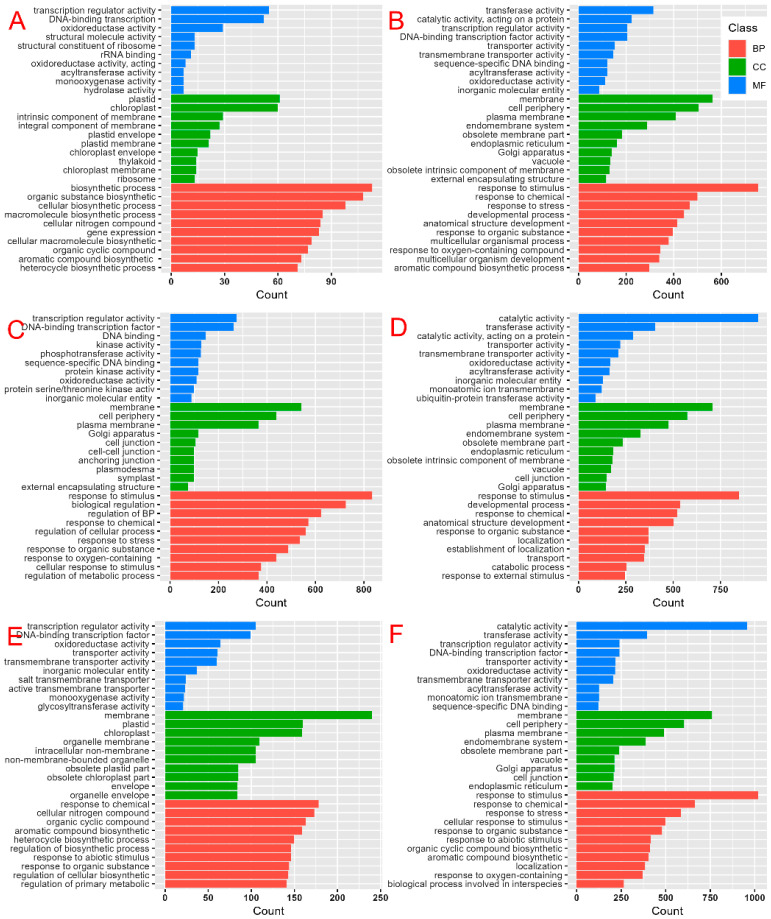
GO terms of DEGs in tobacco. (**A**) Up-regulated DEGs during the small-bud stage. (**B**) Down-regulated DEGs during the small-bud stage. (**C**) Up-regulated DEGs during the medium-bud stage. (**D**) Down-regulated DEGs during the medium-bud stage. (**E**) Up-regulated DEGs during the big-bud stage. (**F**) Down-regulated DEGs during the big-bud stage.

During the three bud stages, 4414, 8134 and 16,269 DEGs were assigned gene ontology (GO) terms in 18 samples. In the small-bud stage, the up-regulated DEGs were primarily involved in the biosynthetic process in the biological process (BP). The enrichment in terms of cell composition (CC) mainly involved the chloroplast. Regarding molecular function (MF), the enrichment was mainly related to transcription regulator activity and oxidoreductase activity (*p* < 0.05) (Figure 5A). Conversely, the down-regulated DEGs were mainly enriched in response to stimuli in BP. The most enriched CC was the membrane, primarily associated with transcription regulator activity and transferase activity in MF (*p* < 0.05) (Figure 5B). In the medium-bud stage, the up-regulated DEGs were mainly enriched in response to stimuli and biological regulation in BP. In CC, they were mainly enriched in the membrane, and in MF, they were mainly enriched in transcription regulator activity (*p* < 0.05) (Figure 5C). Furthermore, the down-regulated DEGs were mainly concentrated in response to stimuli in BP. In CC, they were mainly concentrated in the membrane, and in MF, they were mainly concentrated in catalytic activity and transferase activity (*p* < 0.05) (Figure 5D). In the big-bud stage, the up-regulated DEGs were mainly enriched in the response to chemicals in BP. In CC, they mainly involved the membrane, and in MF, they were mainly concentrated in transcription regulator activity (*p* < 0.05) (Figure 5E). Additionally, the down-regulated DEGs mainly focused on the response to stimuli in BP. In terms of CC, they were mainly concentrated in the membrane and other parts, and in MF, they mainly focused on catalytic activity and transferase activity (*p* < 0.05) (Figure 5F).

**Figure 6:**
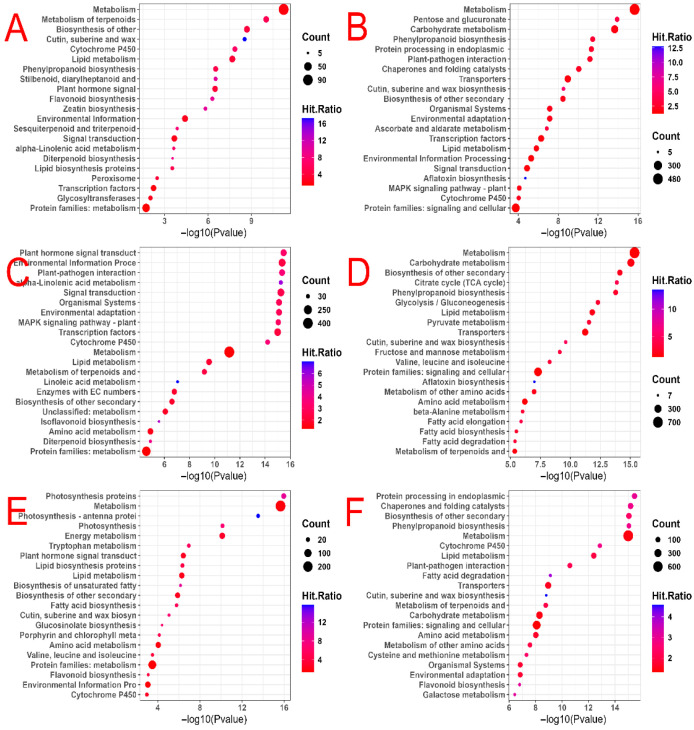
KEGG pathways of DEGs in tobacco. (**A**) Up-regulated DEGs during the small-bud stage. (**B**) Down-regulated DEGs during the small-bud stage. (**C**) Up-regulated DEGs during the medium-bud stage. (**D**) Down-regulated DEGs during the medium-bud stage. (**E**) Up-regulated DEGs during the big-bud stage. (**F**) Down-regulated DEGs during the big-bud stage.

KEGG enrichment analysis revealed that the small-bud stage exhibited significant up-regulation of DEGs primarily involved in metabolism and signal transduction, as well as the plant hormone signal transduction pathway (Figure 6A) (*p* < 0.05). Conversely, down-regulated DEGs were enriched in protein processing in the endoplasmic reticulum and other pathways (*p* < 0.05) (Figure 6B). Moving on to the middle-bud stage, DEGs showed up-regulation and enrichment in signal transduction (*p* < 0.05) (Figure 6C). In contrast, down-regulated DEGs were enriched in pyruvate metabolism and the citrate cycle (TCA cycle) (*p* < 0.05) (Figure 6D). As for in the big-bud stage, DEGs exhibited up-regulation and enrichment in energy metabolism (*p* < 0.05) (Figure 6E). In contrast, down-regulated DEGs were associated with protein processing in endogenous reticulum (*p* < 0.05) (Figure 6F).

### 2.3. The Stamen-Related Gene Dysfunction in CMS K326 Occurred at the Flower Meristematic Tissue Stage

**Figure 7:**
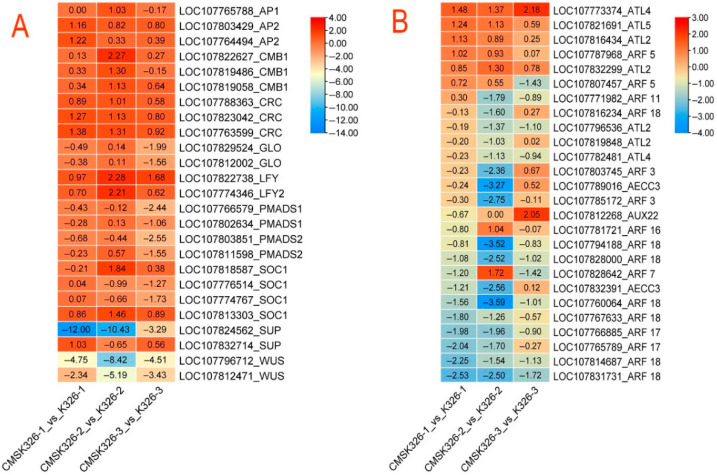
DEGs related to stamen and auxin across all bud stages between CMS K326 and its maintainer. (**A**) DEGs associated with the stamen. (**B**) DEGs associated with auxin. CMS K326-1_vs_K326-1: DEGs in the small-bud stage. CMS K326-2_vs_K326-2: DEGs in the medium-bud stage. CMS K326-3_vs_K326-3: DEGs in the big-bud stage.

The expression of many genes associated with flower development in CMS K326 was abnormal. The expression of *WUSCHEL* (*WUS*) (*LOC107796712*, *LOC107812471*), and *SUP* (*LOC107824562*) in CMS K326 showed a significant decrease in all stages of flower buds compared to the maintainer. Conversely, the expression of *LEAFY* (*LFY*) (*LOC107822738*, *LOC107774346*), *APETALA2* (AP2) (*LOC107803429*, *LOC107764494*) and *CRABS CLAW* (*CRC*) (*LOC107763599*, *LOC107823042*, *LOC107788363*) was increased in CMS K326 at all bud stages (Figure 7A). In CMS K326, the expression of auxin transporter-like protein genes *ATL2* (*LOC107816434*, *LOC107832299*), *ATL4* (*LOC107773374*), *ATL5* (*LOC107821691*) and *MONOPTEROS* (*MP*/*ARF5*) (*LOC107787968*) was up-regulated in every bud stage, unlike the expression of *ARF17* (*LOC107765789*, *LOC107766885*) and *ARF18* (*LOC107831731*, *LOC107828000*, *LOC107767633*, *LOC107814687*) in all bud stages (Figure 7B).

The flower organ develops from the floral meristem and is determined through its initiation and cessation [14]. This process involves many genes, with *WUS* and *AGAMOUS* (*AG*) being particularly important. The inhibition and silence of *WUS* are key to terminating the floral meristem, and *AG* is another essential gene for meristem termination. In *Arabidopsis thaliana*, at stage 3, *LEAFY (LFY)* and *WUS* induce *AG* activity, at the same time; then, *AG* can, in turn, inhibit *WUS*. The expression of *SUP* was observed on both sides of the boundary between the third and fourth whorl carpels at the same stage. Furthermore, *AG* triggered the inhibition of *WUS* by *KNUCKLES* (*KNU*) and *CRC* when the calyx enveloped the bud during stage 6 [14,15]. The floral meristem formed at specific positions within the inflorescence meristem [16], and auxin is a central developmental regulator for flower morphogenesis [17]. Prior to the induction of the flower meristem, there would be a localized concentration peak of auxin at this position. The local maximization of auxin was contingent upon the PIN-FORMED protein [18,19]. The absence of auxin inhibited downstream genes in the auxin signaling pathway by Aux/IAA proteins. When auxin signaling was present, ARF proteins interacted with Aux/IAA proteins and broke them down, which activated downstream genes [20,21]. The central key factor *MP*/*ARF5* in floral meristem development responds to the maximum auxin levels and activates *LFY*. *LFY* then targets *AP1*, thereby regulating the expression of multiple genes involved in floral organ primordia differentiation [22]. The inhibition of auxin biosynthesis by the cadastral gene *SUP* was the key to the termination of meristems after the primordium differentiation of flower organs [15]. Another essential regulator, *AG*, induces the *CRC* of meristem termination by fine-tuning auxin homeostasis [15]. Throughout the entire floral bud development stage, the expression of genes associated with the initiation and cessation of meristematic tissue in CMS K326, such as *WUS*, *LFY*, and *CRC*, exhibited abnormalities. These findings are closely related to the morphological and histological results. The expression of *SUP* also corresponded to the morphological observation of fusion at the base of the stamen and the disappearance of the boundary between the stamen and pistil in the histological observation. These results supported the histological and electron microscope observations that the dysfunction of stamen-related genes in CMS K326 occurred during the meristematic tissue stage. Mutations in *ARF10* [11] and *ARF17* [12] can cause defects in male reproductive tissue, while *ARF18* regulates the specialization of stamen–petal organs in an auxin-dependent manner [23]. The abnormal expression of the auxin transport protein and the auxin response factor genes in CMS K326 at each bud stage showed that auxin disturbance had an essential influence on the floral organ determination of CMS K326.

### 2.4. The Dynamics of Genes Related to Mitochondrial Basic Processes Showed Obstacles

**Figure 8:**
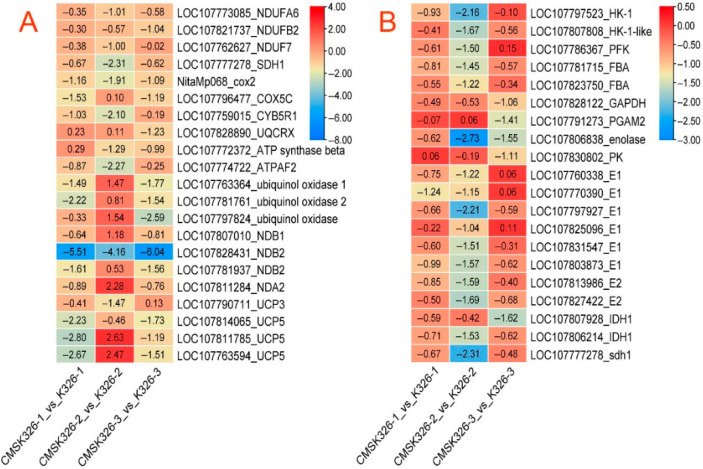
DEGs related to mitochondrial basic processes across all bud stages between CMS K326 and its maintainer. (**A**) DEGs associated with the electron transport chain (ETC). (**B**) DEGs associated with glycolysis and the tricarboxylic acid cycle (TCA). CMS K326-1_vs_K326-1: DEGs in the small-bud stage. CMS K326-2_vs_K326-2: DEGs in the medium-bud stage. CMS K326-3_vs_K326-3: DEGs in the big-bud stage.

The expression of the succinate dehydrogenase flavoprotein subunit 1 (sdh1) (LOC107777278), cytochrome oxidase subunit 2 (cox2) (NitaMp068), and cytochrome b5 reductase 1 (cyb5r1) (LOC107759015) in CMS K326 was down-regulated at every flower bud stage compared to the maintainer. These results suggested abnormalities in the electron transport chain (ETC) of CMS K326 (Figure 8). Additionally, the expression of the ATP synthase mitochondrial F1 complex assembly factor 2-like (ATPAF2) (LOC107774722), NADH dehydrogenase 1 alpha subcomplex subunit 6-like (NDUFA6) (LOC107773085), NADH dehydrogenase 1 beta subcomplex subunit 2-like (NDUFB2) (LOC107821737), NADH dehydrogenase iron-sulfur protein 7 (NDUF7) (LOC107762627), mitochondrial uncoupling protein 5-like (UPC5) (LOC107814065), and external alternative NAD(P)H-ubiquinone oxidoreductase B2 (NDB2) (LOC107828431) also exhibited the same trends in all bud stages. In higher plants, the ETC consists of two branches: the cytochrome oxidase pathway (COP) and the alternative oxidase pathway (AOP), and the results also indicated obstacles in the COP and AOP of CMS K326. Compared with that of its maintainer, the expression of many tricarboxylic acid (TCA) cycle genes in CMS K326 was down-regulated during all bud stages. These genes included pyruvate dehydrogenase E1 component subunit beta-1 (E1) (LOC107831547, LOC107803873), dihydrolipoyllysine-residue acetyltransferase component 1 (E2) (LOC107813986, LOC107827422), and isocitrate dehydrogenase [NAD] regulatory subunit 1 (IDH)(LOC107806214) (Figure 8A). Furthermore, the expression of many essential genes involved in glycolysis was also down-regulated at all bud stages (Figure 8B).

Mitochondria are pivotal in energy conversion and metabolism [1]. Many crop studies have shown that CMS involves mitochondrial genes, such as *atp6*, *atp9*, *nad9*, *cox2*, and *nad7* [4,5,6,8]. The basic mitochondrial processes involve several vital steps, including the substrate entering mitochondria, TCA, oxidative phosphorylation, and ATP synthesis [24,25]. Under aerobic conditions, the TCA completely oxidizes acetyl-CoA into carbon dioxide and water while producing NADH and FADH2. By undergoing oxidative phosphorylation, the chemical energy in NADH and FADH2 was converted into electron flow along the electron transfer chain, leading to the synthesis of ATP. The classical ETC consisted of four different multi-subunit complexes: complex I (NADH ubiquinone oxidoreductase, CI), complex II (succinate dehydrogenase, CII), complex III (cytochrome bc1 oxidoreductase, CIII), and complex IV (cytochrome c oxidase, CIV). If there is a malfunction in the ETC, the NAD(P)H dehydrogenase inside or outside the mitochondrial inner membrane can oxidize succinic acid or NADH. This oxidation process allowed the electrons to be directly supplied to the ubiquinone pool, bypassing complex I without being connected to ATP production [24,25].

In conclusion, the above findings support the existence of obstacles in the mitochondrial energy supply of CMS K326.

## 3. Discussion

While the relationship between CMS and auxin is well-documented, our study uncovers novel mechanisms and phenotypes specific to naturally occurring CMS in tobacco (*Nicotiana tabacum*). Crucially, unlike previously reported cytoplasmic homeosis induced artificially (e.g., via cell fusion or alloplasm), our work reveals the following: (i) natural cytoplasmic homeosis in CMS K326 stamens, suggesting an intrinsic developmental disruption distinct from synthetic systems; (ii) early stamen dysfunction (pre-small-bud stage), implicating developmental timing as a key factor in natural CMS; (iii) mitochondrial ultrastructural defects (swelling, cristae loss, and volume increase) directly linked to floral organ malformation; and coordinated dysregulation of floral development and auxin pathways in transcriptomics, proposing a unified mechanistic basis for natural CMS.

### 3.1. Do Mitochondria Regulate Stamen Development in CMS K326 by Affecting the Auxin Pathway?

The proper expression of genes in plant development relies on nuclear–cytoplasmic coordination, and imbalances in this coordination can disrupt normal growth and development [26]. Moreover, CMS occurs as a result of a disorder in nuclear–cytoplasmic interaction. However, only the stamens in CMS display abnormal characteristics, but their order in the floral whorls remains unchanged [9]. The situation above shows that floral organ identity genes can alter the character of floral organs, but they have a minimal impact on floral organogenesis. The character of the floral organ is determined by floral organ identity genes, which remain expressed throughout most of the floral organ development after being activated [27], so it can be detected at a later stage of flower development.

This study indicates that CMS K326 experienced stamen abnormality during the initial bud stage. Therefore, the abnormality of stamens in CMS results from interfering with the expression of the corresponding stamen organ identity genes during differentiation [28]. When abnormal stamen CMS occurred, the boundary between the stamen in whorl 3 and the pistil in whorl 4 was expected to vanish, the cadastral gene *SUP* was expected to be down-regulated [2]. Research on the *SUP* has shown that abnormal stamens are not the mutual transformation between stamens in whorl 3 and carpels in whorl 4 but the over-proliferation of stem cells in whorl 4 [10]. Stamen abnormality in CMS resulted from the incorrect start and end of the activities of stamen and pistil stem cells during the organogenesis of CMS flowers. The formation of flower meristems heavily relies on the presence of auxin [29], and *SUP* also influences the boundary between whorl 3 and 4 by modulating auxin synthesis [30]. Thus, auxin may be essential in the stamen abnormalities of CMS.

The results show not only that the stamens of CMS K326 were abnormal but also that the boundary between whorl 3 and pistils in whorl 4 disappeared. *WUS*, *SUP*, *ARF10*, and *ARF17* expression decreased, while *AUX* expression increased in all bud stages of CMS K326. The initiation and termination of the meristem depend on the activation and termination of *WUS* [29]. Its activation requires the local maximization of auxin, and *SUP* also acts through auxin [30]. The meristems in N.repanda, sua, and K326 tobacco CMS exhibited sluggish cell division [31]. The *ARF17* mutation also resulted in male reproductive tissue defects [22]. The fertility of tobacco CMS Nta(rep) plants could be partially restored by overexpressing Arabidopsis *SUP* [31]. All evidence supported that mitochondria regulate stamen development in CMS K326 by affecting the auxin pathway.

### 3.2. Are KIN10 and CDKE1 Signal Molecules for the Mitochondrial Retrograde Regulation of Stamen Development?

**Figure 9:**
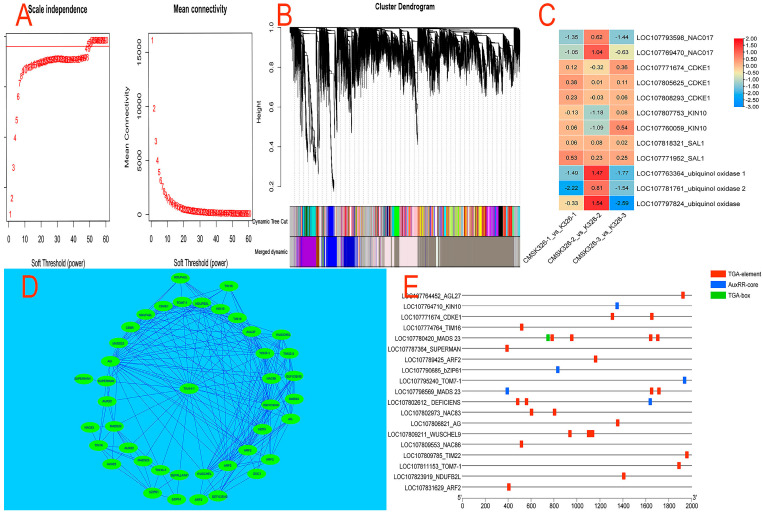
WGCNA’s power value estimation, module, co-expression networks, and hub gene identification between CMS K326 and its maintainer at various bud stages. (**A**) Estimation of the power value. (**B**) Identification of modules. (**C**) Identification of hub genes. (**D**) Co-expression network derived from the antiquewhite1 module. (**E**) Auxin cis-response elements found in several hub genes within the antiquewhite1 module.

WGCNA was used to identify gene clusters co-expressed in three bud stages of CMS K326 (Figure 9). When the power value was set at 45, 51,038 DEGs were grouped into 14 modules, each consisting of 82 to 16,459 genes (Figure 9A,B). The antiquewhite1 module showed a strong correlation with *WUS*, *AG*, and *DEF* among these modules. Through WGCNA, essential genes for flower development (*WUS*, *AG*, *DEF*, *SUP*, *SEP1*), essential mitochondrial ETC genes (*CB5R*, *NDUFA2L*, *NDUFB2*), essential auxin genes (*AUX22*, *ARF2*), mitochondrial outer membrane protein genes (*TOM7-1*), mitochondrial inner membrane protein genes (*TIM14-1*, *TIM16*, *TIM22-2*), and mitochondrial retrograde signaling molecules (*KIN10*, *CDKE1*) were found to be part of the same regulatory module (Figure 9D).

By examining the auxin cis-acting elements upstream of genes, the impact of auxin on various members within the antiquewhite1 module was investigated. Among the module’s 42 members, 19 possessed auxin cis-response elements, which included *WUS*, *AG*, *DEF*, *SUP*, *KIN10*, *CDKE1*, *TIM16*, *TOM7-1*, *TIM22*, *NDUFB2*, and others. Auxin cis-response elements were categorized as AuxRR, TGA-element, and TGA-box types. The most significant number of cis-response elements, a total of five, was found in *MADS23*, surpassing all other members. Only one AuxRR was present in *KIN10*, *bZIP61*, and *TOM7-1*, whereas *MADS23* and *DEF* exhibited both AuxRR and TGA elements. The remaining genes primarily had TGA elements, four and three cis-response elements in *WUS* and *DEF*, respectively (Figure 9E).

Any abnormal phenotypes in CMS should stem from changes in the expression of nuclear genes controlled by mitochondria. This, in turn, causes specific target genes to contribute indirectly to male sterility. According to studies, *AOX1a*, *ANAC017*, *KIN10*, and *CDKE1* were confirmed as mitochondrial retrograde regulatory molecules, with *ANAC017* being crucial for *AOX1a* activation [32,33]. *ANAC017*, *KIN10*, and *CDKE1* have been detected in CMS K326, and their expression has also been observed to be abnormal. Notably, *AOX1a* was absent in tobacco, but a gene homologous to ubiquinone oxidase was present.

In CMS K326, the expressions of *NAC017* (LOC107769470, LOC107793598), *ubiquinol oxidase* (LOC107797824), *ubiquinol oxidase 1* (LOC107763364), and *ubiquinol oxidase 2* (LOC107781761) were down-regulated in the small- and big-bud stages and up-regulated in the medium-bud stage. *KIN10* expression was reduced during the medium-bud stage, whereas *CDKE1* expression exhibited minor fluctuations across all bud stages (Figure 9C).

The WGCNA indicated that *KIN10* and *CDKE1* were part of the same module as flower development genes *WUS*, *AG*, *DEF*, *SUP*, and *SEP1*; auxin-related genes *AUX22* and *ARF2*; and other significant genes, *CB5R*, *NDUFA2L*, and *NDUFB2L*, in ETC. It is worth mentioning that *WUS*, *AG*, *DEF*, *SUP*, *AUX22*, *ARF2*, *NDUFB2L*, *KIN10*, and *CDKE1* all contain auxin response elements. As a result, it was hypothesized that *KIN10* and *CDKE1* might function as retrogressive regulators of CMS K326 stamen development. Unfortunately, this module does not contain ubiquinone oxidase and *NAC017*. It is intriguing to question whether ubiquinone oxidase and *ANAC017* are not involved in the retrograde regulation of tobacco CMS K326 or if they indirectly affect this module through other means.

## 4. Materials and Methods

### 4.1. Materials

CMS and maintainer K326 plants were cultivated in an experimental field at Jiangxi Agricultural University, Nanchang, China (28°45′54″ N, 115°50′1″ E). CMS K326 was developed from a naturally sterile plant of the tobacco variety ‘Tiegu’ (*N. tabacum*), which had undergone 33 generations of continuous backcrossing with flue-cured tobacco variety K326. The stamens of CMS K326 showed abnormal polymorphism, and sterility was stable in different environments.

Floral buds were imaged at three developmental stages: small bud (<3 mm), medium bud (3–5 mm), and big bud (>5 mm). After removing sepals and petals, samples were imaged using an Optec SZ780 stereo microscope equipped with a 5MP CMOS camera (Optec, Chongqing, China). Images were acquired under uniform bright-field illumination at 6.7×–45× magnification, with scale bars calibrated using a stage micrometer.

### 4.2. Histological Analysis

The conventional paraffin sectioning method was implemented according to Farbos [34]. The samples were fixed with 50% FAA and then rinsed with distilled water and dehydrated using a series of alcohol gradients, with each gradient applied for 15 min. After dehydration, the samples were cleared in an alcohol–xylene solution and then embedded in paraffin, and tissue sections of 6–9 μm in thickness were obtained, mounted on gelatin-coated slides, and dried in a drying chamber. After dewaxing with xylene, the sections were stained with 0.5% safranin solution, followed by counterstaining with 1% fast green solution. After dehydration and clearing, the sections were cover-slipped using a neutral mounting medium and then observed and photographed using a Zeiss fluorescence microscope.

### 4.3. Electron Microscopy Observation

Sample preparation followed the high-pressure freezing and freeze-substitution protocol of Hayes et al. [35] with modifications. Undifferentiated floral buds (pistil and stamen primordia < 0.7 mm) were selected using a Leica M80 stereomicroscope (Leica Microsystems, Wetzlar, Germany) and immediately frozen using a Leica HPM100 high-pressure freezer (Leica Microsystems).

For freeze substitution, samples were immersed in anhydrous acetone containing 1% osmium tetroxide (OsO_4_) and 0.2% uranyl acetate (UA) at −85 °C for 72 h in a Leica EM AFS2 freeze substitution system (Leica Microsystems). The temperature was gradually raised to 4° C over 8 h before resin infiltration.

Samples were infiltrated with Poly/Bed 812 epoxy resin (Polysciences, Warrington, PA, USA) through a graded series (30%, 50%, 70%, 100%) over 48 h and polymerized at 60° C for 48 h. Ultrathin sections (70 nm) were cut using a Leica EM UC7 ultramicrotome (Leica Microsystems) with a 35° diamond knife (Diatome, Nidau, Switzerland).

Post-staining was performed sequentially with (i) 2% uranyl acetate in 50% ethanol (30 min) and (ii) Reynolds’ lead citrate (5 min).

Sections were examined using a Hitachi HT7800 transmission electron microscope (Hitachi High-Technologies, Tokyo, Japan) operating at 80 kV. Images were acquired with an EMSIS Morada G3 CCD camera (EMSIS GmbH, Münster, Germany) using iTEM Control Software (v5.2) at magnifications ranging from 2000× to 50,000×.

### 4.4. RNA Extraction, Sequencing, and Bioinformatics Analysis

The flower buds of CMS and maintainer K326 in three developmental stages (<3 mm, 3–5 mm, and >5 mm) were sampled and immediately frozen at −80 °C using liquid nitrogen for RNA extraction. Three biological replicates of each sample were obtained, with each containing more than 30 buds.

Total RNA was extracted using an RNA Extraction Kit (Zhongke New Life, Shanghai, China). After sample qualification, the mRNA was enriched with magnetic beads containing Oligo(dT). The first cDNA strand was synthesized using mRNA as a template and six-base random hexamers. DNA polymerase I, buffer, and dNTPs were added to synthesize the second cDNA strand, and the double-stranded cDNA was then purified using AMPure XP beads. The fragment size was selected with AMPure XP beads, and PCR enrichment was performed to obtain the final cDNA library. Once the library inspection was complete, NGS was implemented using the Illumina HiSeqTM 2000 platform (Illumina, San Diego, CA, USA).

Any reads with base mass values of 25 or lower and N ratios exceeding 5% were removed. The clean reads were aligned to the TN90 tobacco genome (Available online: https://ftp.ncbi.nlm.nih.gov/genomes/refseq/plant/Nicotiana_tabacum/all_assembly_versions/GCF_000715075.1_ASM71507v2/ (accessed on 20 August 2025)) using HISAT2 [36] to obtain their positional information. To determine the expression levels of each gene in every sample, featureCounts [37] was employed, with the results denoted as reads per kilobase of the exon model per million mapped reads (FPKM). DESeq2 [38] was employed for gene differential expression analysis, and the *p*-values generated from the original hypothesis testing were adjusted. Genes with a *p*-value below 0.05 and an absolute log2foldchange greater than 1 were classified as significantly differentially expressed genes (DEGs). Next, the blast algorithm was used to annotate the obtained sequences against the NR database (Available online: https://ftp.ncbi.nlm.nih.gov/blast/db/FASTA/, accessed on 20 August 2025).

KEGG pathway enrichment analysis was conducted using R (version 4.4.2) with the clusterProfiler package (version 4.14.6) (Yu et al., 2012) [39]. The enriched pathways were visualized using ggplot2 and enrichplot packages.

### 4.5. Weighted Gene Correlation Network Analysis (WGCNA)

All samples were analyzed using WGCNA [40] to gain further insights into the interactions among differentially expressed genes (DEGs). Genes with counts below 60 across all samples were removed, and the top 90% of genes, based on variance, were selected for the WGCNA. The analysis was conducted using a recommended stepwise method, with a minimum of 30 modular genes. The clustering method identified highly similar modules, which were then represented using different colors. The process involved clustering to identify these similar modules, after which the new modules were merged based on their characteristic genes. The MCODE tool was used to screen for hub genes, and the co-expression network was visualized using Cytoscape (v3.10.1) [41]. TBtools(v2.326) [42] was used to extract the upstream 2 kb sequences of hub genes based on the tobacco gene annotation, and the upstream cis-response elements in the gene promoters were analyzed using PlantCare (Available online: http://bioinformatics.psb.ugent.be/webtools/plantcare/html/, accessed on 20 August 2025) [43].

### 4.6. Reverse Transcription Pcr (Rt-Pcr) Validation

We selected 21 genes at random to validate the accuracy of the transcriptome data with RT-PCR. RNA was extracted from the floral buds of CMS K326 and its maintainer in triplicate, and reverse transcription amplification was performed with a Goldenstar^®^ RT6 cDNA Synthesis Kit (Tsingke, Beijing, China). The cDNA products generated in this manner were diluted appropriately and used as a qPCR template for amplification with 2 × T5 Fast qPCR Mix (SYBR Green I) (Tsingke, Beijing, China). The experiment was performed according to the instructions, and three biological replicates were obtained for each sample. The primers were designed with Oligo 7.0 [44] (Table S1), and the relative quantitative results of the genes were calculated using the 2^−△△Ct^ method based on the Ct values. The difference in expression was calculated according to the amplification efficiency of 100% [45].

## 5. Conclusions

In this study, morphology, tissue section analysis, and electron microscope observation not only confirmed the distinction between CMS K326 and its maintainer during the flower meristem stage but also revealed that the interaction between the nucleus and cytoplasm results in the polymorphism of the stamens. Analysis of DEGs and WGCNA suggests that mitochondria might regulate the stamen development of CMSK326 through the auxin pathway. In this study, we used only transcriptome sequencing at three different bud stages, but we did not precisely detect transcriptomic characteristics during the floral meristem phase or measure auxin concentrations in different tissues. Resolution and accuracy could be enhanced through precise sampling of the floral meristem and measurement of auxin concentrations across various floral developmental stages in different tissues. Importantly, additional methods and efforts are required to validate the roles of key candidate genes in CMS. In summary, in this research, we propose a potential pathway for CMS in tobacco, with the findings revealing the complex interplay between mitochondria and stamen morphology in the occurrence of CMS in tobacco.

## Figures and Tables

**Table 1:** Summary of transcriptome sequencing data generated for 18 samples.

Sample	Raw Reads	Clean Reads	Clean Bases (G)	Q30 (%) ^a^	Total Mapped	Non-Splice Reads (%)	Splice Reads (%)	GC (%)
CK-K326-1-1	104,512,128	103,875,880	12.91	94.43	99,966,911 (96.24%)	59.14	30.39	42.18
CK-K326-1-2	102,055,434	101,280,986	12.69	94.23	97,383,205 (96.15%)	58.99	30.64	42.2
CK-K326-1-3	99,866,614	99,078,916	12.47	94.5	95,337,710 (96.22%)	58.93	30.87	42.18
CK-K326-2-1	104,166,470	103,170,638	12.69	94.22	99,229,123 (96.18%)	61.68	28.16	41.9
CK-K326-2-2	103,706,794	102,812,584	12.68	94.19	98,947,284 (96.24%)	61.67	28.29	41.95
CK-K326-2-3	106,884,174	105,948,224	13	94.35	101,884,105 (96.16%)	61.77	28.03	41.84
CK-K326-3-1	103,375,706	102,648,638	13.33	94.26	98,891,972 (96.34%)	59.86	30.94	42.5
CK-K326-3-2	84,662,664	84,101,954	10.89	94.26	81,019,601 (96.33%)	59.96	30.78	42.5
CK-K326-3-3	90,994,404	90,383,882	11.6	94.62	87,084,579 (96.35%)	60.24	30.35	42.39
SK326-1-1	136,226,838	134,977,386	15.62	94.51	128,613,155 (95.28%)	59.46	26.82	41.98
SK326-1-2	108,540,094	107,363,826	12.82	94.21	102,478,800 (95.45%)	59.22	27.91	42.06
SK326-1-3	114,793,874	113,187,096	13.55	94.48	108,027,225 (95.44%)	59.19	27.97	41.98
SK326-2-1	92,449,656	91,199,248	11.22	94.27	87,141,096 (95.55%)	58.75	28.86	42.24
SK326-2-2	94,939,256	93,649,454	11.35	94.27	89,419,575 (95.48%)	58.98	28.31	42.16
SK326-2-3	96,782,486	95,665,092	11.47	94.44	91,305,843 (95.44%)	59.02	27.98	42.16
SK326-3-1	106,661,546	105,373,566	12.44	94.3	100,422,379 (95.30%)	59.07	27.43	42.21
SK326-3-2	106,576,346	105,247,334	12.42	94.28	100,219,624 (95.22%)	59.19	27.27	42.13
SK326-3-3	108,111,576	106,703,754	12.52	94.67	101,667,123 (95.28%)	59.35	27.08	42.07

^a^ Average of data collected from three biological replications. Mapped reads: reads that were uniquely mapped onto the tobacco reference genome.

## Data Availability

Data will be made available on request. The sequencing data have been deposited in the Sequence Read Archive at the National Center for Biotechnology Information (NCBI) with the BioProject ID PRJNA795655 (Available online: https://www.ncbi.nlm.nih.gov/bioproject/PRJNA795655, accessed on 20 August 2025).

## Supplementary Materials

The following supporting information can be downloaded at https://www.mdpi.com/article/10.3390/plants14172613/s1, Table S1. Genes and their primers used in RT-PCR; Figure S1. Validation of DEGs by RT-PCR. Figure S2. Mitochondria in the meristematic cells of floral buds from the maintainer line K326. Figure S3. Mitochondria in the meristematic cells of floral buds from CMS K326 (original image of Figure 3B).

## Abbreviations

The following abbreviations are used in this manuscript:

CMS	Cytoplasmic Male Sterility
ETC	Electronic Transport Chain
WGCNA	Weighted Gene Correlation Network Analysis
DEG	Differentially Expressed Gene
TCA	Tricarboxylic Acid Cycle
