# Supplementary material for: Integrated Histological, Ultrastructural, and Transcriptomic Analyses Reveal New Insights into Stamen Development in Cytoplasmic Male Sterile Tobacco (CMS K326)
Source: Plants (Basel). 2025 Aug 22;14(17):2613. doi: 10.3390/plants14172613 (PMC12430244; doi:10.3390/plants14172613)
Supplement: Supplementary file 1 [file plants-14-02613-s001.zip › plants-3764595-supplementary.pdf]

Table S1. Genes and their primers used in RT-PCR.

Gene	Forward Primer (5′–3′)	Reverse Primer (5′–3′)	Annotation
LOC107781937	GCAACAGCAAATCTCAGGTGAA	TTGCTGCCTCAGAGTATGCC	*NDB2*
LOC107791218	CCGCGTCATAGAGAGAAAGGG	TTAGTAATTCTGCTGCTGCTGCT	*SVG*
LOC107809546	AGGTGAAGGAGTGAGCTCGT	TCCTTCATACCAAACACACCCT	*AGL11*
LOC107810002	GCGTTTGCCGTTTCCAGAAT	GGGGGTGGGGAATTTTTATGTG	*AGL15*
LOC107767389	GCAAAGCGCAGTCTGATTGA	CGCATCTTTTGGCCAACTCC	*AGL61*
LOC107828933	AAGTGACGTTCTCGAAGCGG	CCCGTCTTCATCATGCCCTC	*AGL62*
LOC107763364	AGATGATGACACGTGGAGCG	CGCCACTCATAACTCCGGTT	*AOX1A*
LOC107764494	GACCTGAGCTTGGGTGGTTC	AGGCCTTAATCCTTGCTGCC	*AP2*
LOC107803429	TTGGTGTCCAGGTTCGTTCC	CTTTGGCATCTCGCTTGACG	*AP2*
LOC107760064	TTCTGATGAACCCAAGCAGC	AGACCAGTGATCCATTTCCTGA	*ARF10*
LOC107763599	TGTCTGTTTCCTGCTATAACCCT	GACTAGTATATGGGCAGAGAGCA	*CRC*
LOC107823042	GCATGCATGAAGTGGTTGGAA	GGCCTTGGAGTGGAGGTCTA	*CRC*
LOC107800137	GTGCCGGAGACTCAGAAAGT	GCTGCCACACAGTACATTCC	*CUC2*
LOC107807098	CAACAACCCAGCAGCAGTATT	CATCTTGAGGAGGAGGGCCA	*JOINTLESS*
LOC107764171	AAATGGGGGAGTTTGAGCCA	TTTGCGCCCACAGGATGATT	*NAC017*
LOC107768182	AGCTGTGAGGTAATTTCTAAGGG	ACATTCAAGCTGCAACCCAAG	*SOC1*
LOC107824562	CCAGAACCATGTTGGAGCGG	TCACACGCCAATCATCTCCG	*SUP*
LOC107832714	CAGCCAGCAGATTTTGCCAAG	CAAGGCGAAGTTCCAAATCCA	*SUP*
LOC107764083	AGTTTCAAGCAAGCAAGGGC	TGCTCGAAGAAGAGAAGGCG	*TOM20-3*
LOC107796712	TCCTCACTCTTCAGTCTTCAGTC	TGGTTTTGTTGTTGGGCAGC	*WUS*

LOC10781247 1	TGGCAGATCTCCTAATTCCCC	TACCATGGTCCATAGGCACG	*WUS*

Figure S1. Validation of DEGs by RT-PCR.

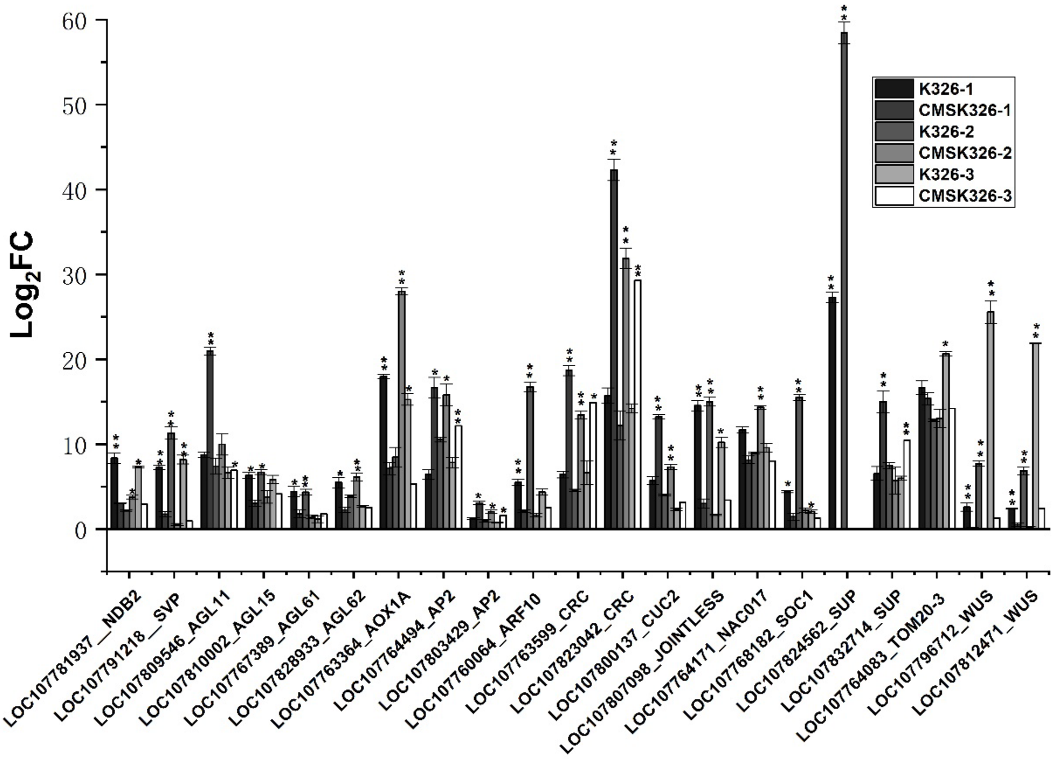

Note: Significance was analyzed using Student's t test, and Error bars show the SD (*n*=3). All data are shown as means ± standard deviations (SDs) (*n*=3). **significant at *P*=0.01, *significant at *P*=0.05.

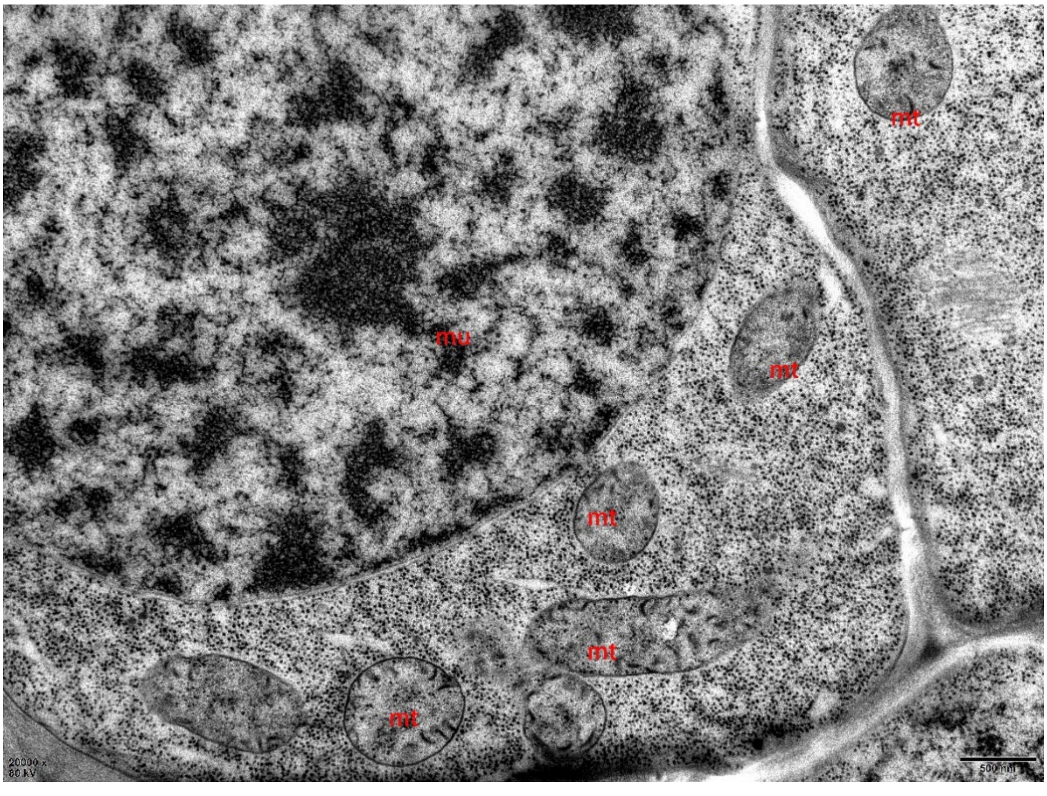

Figure S2. Mitochondria in the meristematic cells of floral buds from the maintainer line K326. The image shows mitochondrial morphology, including both transverse and longitudinal sections of mitochondria, with clearly arranged and distinct cristae. The scale bar (500 nm) was automatically added during imaging.nu: nucleus. mt: mitochondria.

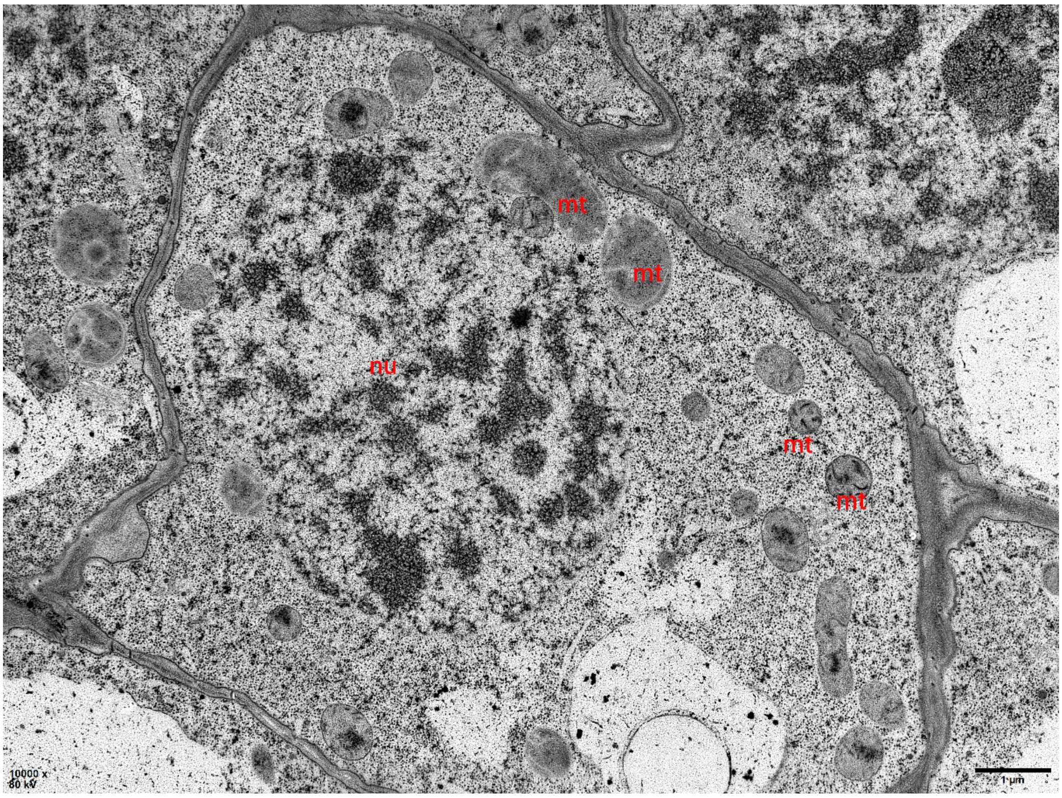

Figure 3B). The image shows two distinct types of mitochondria: normal morphology (lower right), similar to those observed in the maintainer line K326, with clearly visible but irregularly arranged cristae; swollen morphology (upper right), exhibiting significant volume expansion, indistinct cristae, and disorganized structure. The scale bar (1 μm) was automatically added during imaging. nu: nucleus. mt: mitochondria.
